# ICTV Virus Taxonomy Profile: *Herpesviridae* 2021

**DOI:** 10.1099/jgv.0.001673

**Published:** 2021-10-27

**Authors:** Derek Gatherer, Daniel P. Depledge, Carol A. Hartley, Moriah L. Szpara, Paola K. Vaz, Mária Benkő, Curtis R. Brandt, Neil A. Bryant, Akbar Dastjerdi, Andor Doszpoly, Ursula A. Gompels, Naoki Inoue, Keith W. Jarosinski, Rajeev Kaul, Vincent Lacoste, Peter Norberg, Francesco C. Origgi, Richard J. Orton, Philip E. Pellett, D. Scott Schmid, Stephen J. Spatz, James P. Stewart, Jakob Trimpert, Thomas B. Waltzek, Andrew J. Davison, ICTV Report Consortium

**Affiliations:** ^1^​ Lancaster University, UK; ^2^​ NYU School of Medicine, New York, New York, USA; ^3^​ The University of Melbourne, Victoria, Australia; ^4^​ Pennsylvania State University, Pennsylvania, USA; ^5^​ Veterinary Medical Research Institute, Eötvös Loránd Research Network, Budapest, Hungary; ^6^​ University of Wisconsin-Madison, Madison, Wisconsin, USA; ^7^​ University of Cambridge, UK; ^8^​ Animal and Plant Health Agency-Weybridge, Addlestone, Surrey, UK; ^9^​ Virokine Therapeutics, London BioScience Innovation Centre, Royal Veterinary College, London, UK; ^10^​ Gifu Pharmaceutical University, Gifu, Japan; ^11^​ University of Illinois at Urbana-Champaign, Urbana, Illinois, USA; ^12^​ University of Delhi South Campus, New Delhi, India; ^13^​ Institut Pasteur du Laos, Vientiane, Lao PDR, Laos; ^14^​ University of Gothenburg, Gothenburg, Sweden; ^15^​ University of Bern, Switzerland; ^16^​ University of Glasgow, UK; ^17^​ Wayne State University School of Medicine, Detroit, Michigan, USA; ^18^​ Centers for Disease Control and Prevention, Atlanta, Georgia, USA; ^19^​ US National Poultry Research Center, Athens, Georgia, USA; ^20^​ University of Liverpool, UK; ^21^​ Freie Universität Berlin, Berlin, Germany; ^22^​ University of Florida, Gainesville, Florida, USA

**Keywords:** *Herpesviridae*, ICTV Report, taxonomy

## Abstract

Members of the family *Herpesviridae* have enveloped, spherical virions with characteristic complex structures consisting of symmetrical and non-symmetrical components. The linear, double-stranded DNA genomes of 125–241 kbp contain 70–170 genes, of which 43 have been inherited from an ancestral herpesvirus. In general, herpesviruses have coevolved with and are highly adapted to their hosts, which comprise many mammalian, avian and reptilian species. Following primary infection, they are able to establish lifelong latent infection, during which there is limited viral gene expression. Severe disease is usually observed only in the foetus, the very young, the immunocompromised or following infection of an alternative host. This is a summary of the International Committee on Taxonomy of Viruses (ICTV) Report on the family *Herpesviridae*, which is available at ictv.global/report/herpesviridae.

## Virion

**Fig. 1.**
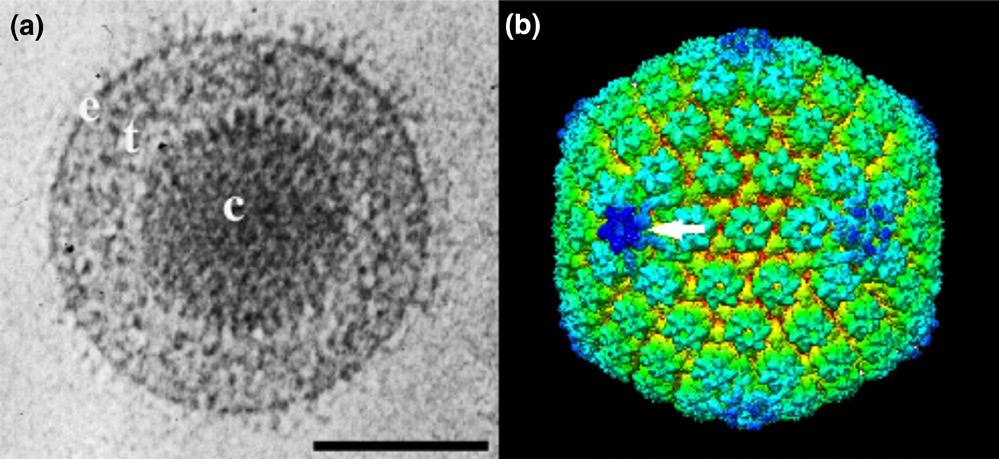
Herpes simplex virus type 1 virion and capsid structure. (**a**) Electron cryo-microscopic image of a virion showing the capsid (**c**), tegument (**t**) and envelope (**e**). Scale bar, 100 nm. From [2] with permission. (**b**) Three-dimensional image reconstruction of a capsid showing hexons, pentons and the portal (arrow). From [5].

Virions consist of a core, capsid, tegument and envelope (Table 1, Fig. 1) [1]. The core comprises the viral genome packaged into the capsid as a linear, dsDNA molecule. The capsid is a *T*=16 icosahedron containing 162 capsomers arranged as 150 hexons, 11 pentons and one portal. The tegument consists of inner and outer layers. The lipid envelope contains integral viral glycoproteins forming a network of spikes.

**Table 1.** Characteristics of members of the family *Herpesviridae*

Example:	herpes simplex virus type 1 (JN555585), species *Human alphaherpesvirus 1*, genus *Simplexvirus*, subfamily *Alphaherpesvirinae*
Virion	Spherical (150–200 nm) particles with condensed DNA core, icosahedral capsid, tegument and a lipid envelope containing glycoproteins
Genome	125–241 kbp of linear dsDNA
Replication	Infection has lytic and latent phases; transcription occurs in the nucleus by a kinetic cascade; DNA replicates by a rolling-circle mechanism to generate concatemers, from which genomes are cleaved and packaged into preformed capsids; virions mature in the cytoplasm
Translation	Occurs from capped, polyadenylated mRNAs, some of which are spliced
Host range	Mammals, birds and reptiles
Taxonomy	Realm *Duplodnaviria*, kingdom *Heunggongvirae*, phylum *Peploviricota*, class *Herviviricetes*, order *Herpesvirales*; 3 subfamilies, >10 genera and >100 species

## Genome

The genome is 125–241 kbp. The arrangement of direct or inverted repeats at the termini or internally results in several classes of genome architecture. The genome contains 70–170 genes encoding proteins, 43 of which are shared across the family, suggesting a common replication strategy [2]. Additional genes encoding nontranslated RNAs may be present.

## Replication

**Fig. 2.**
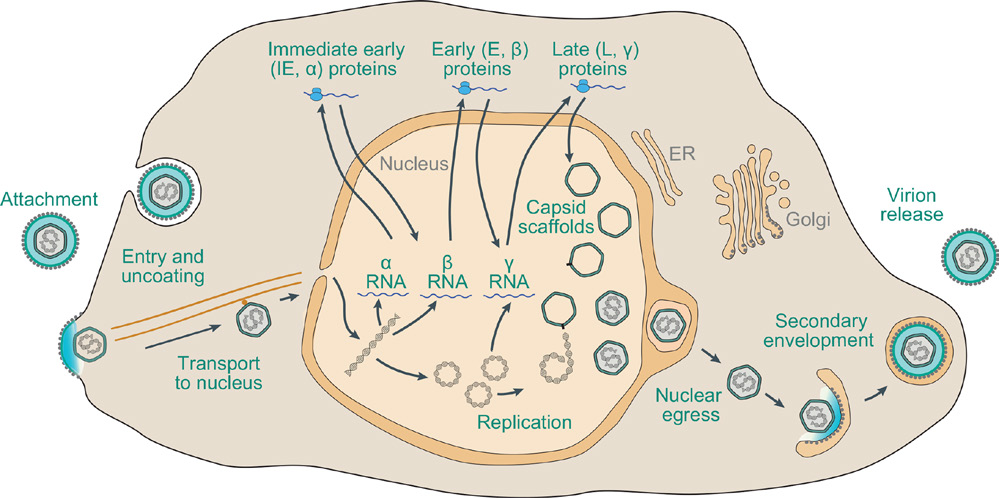
Schematic representation of the lytic replication cycle of a representative herpesvirus in permissive cells.

Herpesviruses have been discovered in a wide range of vertebrates (reptiles, birds and mammals). The most extensively studied animals are host to members of several species. Most herpesviruses have coevolved and sometimes cospeciated within a single host lineage, although foundational cross-species transmission events appear to have occurred. In general, lytic infection involves attachment and penetration by the interaction of virion envelope proteins with cell surface receptors, followed by entry via membrane fusion at the cell surface or after endocytosis (Fig. 2) [3, 4]. The capsid uncoats and is transported to a nuclear pore, and the genome enters the nucleus. Transcription occurs in a kinetic cascade: immediate early genes encode regulatory functions, early genes encode the DNA replication complex and a variety of proteins involved in modifying host cell metabolism or immune responses, and late genes primarily encode virion proteins. Viral DNA synthesis occurs by a rolling-circle replication mechanism to generate concatemers, from which genomes are cleaved and packaged into capsids. Capsids bud through the inner nuclear membrane and are then de-enveloped by fusion with the outer nuclear membrane and released into the cytoplasm. Assembly of tegument proteins and secondary envelopment to generate mature virions occur in a Golgi or post-Golgi compartment. Virions exit the cell by exocytosis or cell-to-cell spread.

Herpesviruses are restricted in their natural host range and highly adapted to their hosts, with severe infection usually observed only in the foetus, the very young, the immunocompromised or in an alternative host. Typically, a primary, systemic infection is established via a cell-associated viraemia, followed by a latent phase in which dormant virus occasionally reactivates. Herpesviruses operate a range of modulation mechanisms to manage host immunity.

## Taxonomy

Current taxonomy: ictv.global/taxonomy. The family *Herpesviridae* includes three subfamilies, and belongs to the order *Herpesvirales* along with the families *Alloherpesviridae* and *Malacoherpesviridae*. This order is in turn classified alongside the order *Caudovirales* in the kingdom *Heunggongvirae*.

## Resources

Current ICTV Report on the family *Herpesviridae*: ictv.global/report/herpesviridae.
